# Incidence and risk factors of postoperative delirium following hepatic resection: a retrospective national inpatient sample database study

**DOI:** 10.1186/s12893-024-02436-w

**Published:** 2024-05-14

**Authors:** Rui Liu, Ningyuan Liu, Shanlian Suo, Qinfeng Yang, Zhen Deng, Wei Fu, Min Wang

**Affiliations:** 1 Department of Anesthesiology, The Second Hospital of Lanzhou University, Lanzhou, Gansu 730030 China; 2 The First School of Clinical Medicine, Southern Medical University, Guangzhou, Guangdong 510515 China; 3 Division of Orthopaedic Surgery, Department of Orthopaedics, Nanfang Hospital, Southern Medical University, Guangzhou, Guangdong 510515 China

**Keywords:** Hepatic resection, Postoperative delirium, Complications, Comorbidities

## Abstract

**Background:**

Postoperative delirium (POD) is a common complication after major surgery and can cause a variety of adverse effects. However, no large-scale national database was used to assess the occurrence and factors associated with postoperative delirium (POD) following hepatic resection.

**Methods:**

Patients who underwent hepatic resection from 2015 to 2019 were screened using the International Classification of Diseases (ICD) 10th edition clinical modification code from the National Inpatient Sample (NIS) Database. Peri-operative factors associated with delirium were screened and underwent statistical analysis to identify independent predictors for delirium following hepatic resection.

**Results:**

A total of 80,070 patients underwent hepatic resection over a five-year period from 2015 to 2019. The overall occurrence of POD after hepatic resection was 1.46% (1039 cases), with a slight upward trend every year. The incidence of elective admission was 6.66% lower (88.60% vs. 81.94%) than that of patients without POD after hepatic resection and 2.34% (45.53% vs. 43.19%) higher than that of patients without POD in teaching hospitals (*P* < 0.001). In addition, POD patients were 6 years older (67 vs. 61 years) and comprised 9.27% (56.69% vs. 47.42%) more male patients (*P* < 0.001) compared to the unaffected population. In addition, the occurrence of POD was associated with longer hospitalization duration (13 vs. 5 days; *P* < 0.001), higher total cost ($1,481,89 vs. $683,90; *P* < 0.001), and higher in-hospital mortality (12.61% vs. 4.11%; *P* < 0.001). Multivariate logistic regression identified hepatic resection-independent risk factors for POD, including non-elective hospital admission, teaching hospital, older age, male sex, depression, fluid and electrolyte disorders, coagulopathy, other neurological disorders, psychoses, and weight loss. In addition, the POD after hepatic resection has been associated with sepsis, dementia, urinary retention, gastrointestinal complications, acute renal failure, pneumonia, continuous invasive mechanical ventilation, blood transfusion, respiratory failure, and wound dehiscence / non-healing.

**Conclusion:**

Although the occurrence of POD after hepatic resection is relatively low, it is beneficial to investigate factors predisposing to POD to allow optimal care management and improve the outcomes of this patient population.

## Background

Most people with liver cancer have liver dysfunction caused by other liver diseases, alcoholism, or hepatitis virus infections [1]. In the United States, hepatic resection is increasingly used for resection of biliary tract malignancies, primary liver cancer, and metastases [2]. Major hepatic resection is an extremely risky procedure, especially in cases with poor liver condition, with complications occurring after surgery in 41-50% of patients [3–5]. It is now understood that there is a high risk of postoperative delirium (POD) after hepatic resection [6].

POD is a multifactorial and heterogeneous syndrome defined as an acute alteration of consciousness associated with an underlying physiological disorder [7, 8]. POD is common postoperatively, with the incidence of POD varying between 4% and 61%, depending on the type of surgery [9–11]. The occurrence of POD leads to higher complication rates, longer recovery times, additional care, prolonged intensive care unit (ICU) stays, and greater costs [12, 13]. Although the pathophysiology of POD is unknown, risk factors for POD include the patient’s age, drug-related factors, medical condition, postoperative analgesic regimen, and surgical factors [14–16].

Recently, it has been reported that POD after hepatic resection in the elderly is associated with serum albumin concentration and age [6, 17, 18]. However, to date, few studies have elucidated POD in patients undergoing hepatic resection. Therefore, it is important to investigate the factors that contribute to POD in hepatic resection cases and identify factors that increase the risk of developing POD. This will provide a theoretical basis for the prevention of POD. Herein, the clinical data of hepatic resection inpatients were retrospectively analyzed with the aim of providing an overview of the frequency of occurrence of POD events in this patient cohort and an in-depth study of potential risk factors associated with POD.

## Methods

### Data source

The National Inpatient Sample (NIS) database, conducted by the Healthcare Cost and Utilization Project and sponsored by the Agency for Healthcare Research and Quality, provided data for your research. In the United States, NIS is the largest database of all payers for hospitalization admissions. NIS collects stratified samples from more than 1,000 hospitals, accounting for about 20% of annual hospitalizations in the U.S [19]. Data collected from the NIS database, including hospital characteristics, type of insurance, patient demographics, length of stay (LOS), in-hospital mortality, total cost, and diagnostic and procedure codes for the International Classification of Diseases (10th Revision) Clinical Revision (ICD-10-CM). This study utilized anonymized data from public sources and is thus classified as exempt from requiring ethical approval according to established research guidelines.

### Data collection

**Fig. 1:**
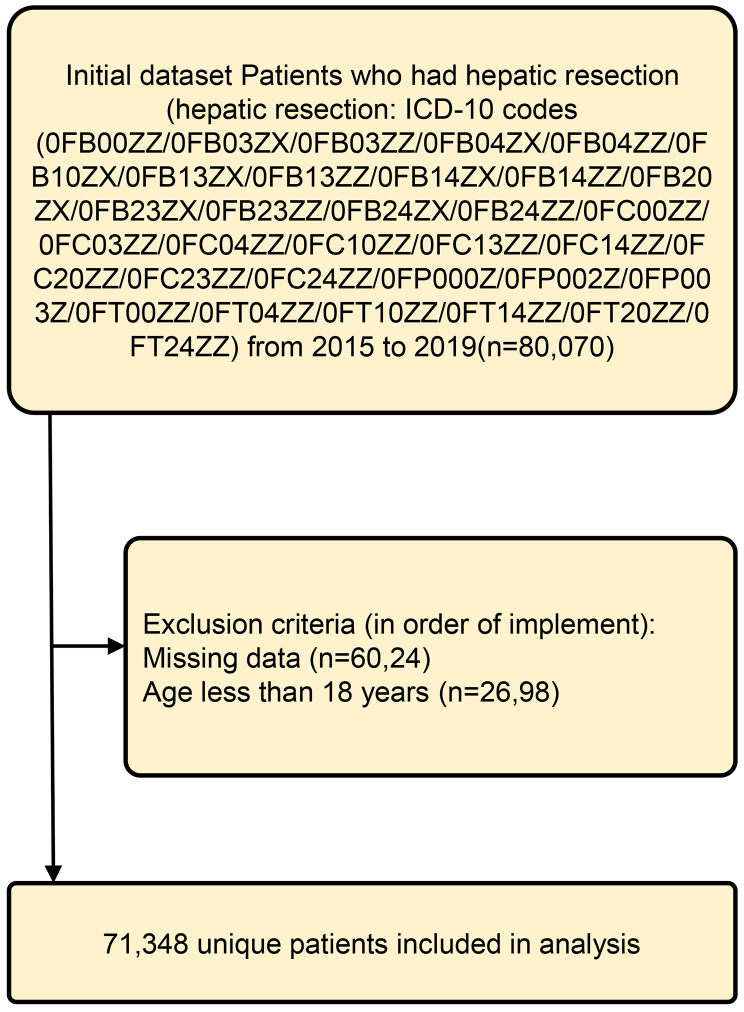
Flow diagram of study selection process. ICD-10, International Classification of Diseases (Tenth Revision) Clinical Modification

The cohort under investigation comprised individuals for whom hospitalization information was accessible in the National Inpatient Sample database spanning the years 2015 to 2019. The focus was on patients undergoing hepatic resection, identified through specific ICD-10-CM procedural codes: 0FB00ZZ, 0FB03ZX, 0FB03ZZ, 0FB04ZX, 0FB04ZZ, 0FB10ZX, 0FB13ZX, 0FB13ZZ, 0FB14ZX, 0FB14ZZ, 0FB20ZX, 0FB23ZX, 0FB23ZZ, 0FB24Z, 0FB24ZZ, 0FC00ZZ, 0FC03ZZ, 0FC04ZZ, 0FC10ZZ, 0FC13ZZ, 0FC14ZZ, 0FC20ZZ, 0FC23ZZ, 0FC24ZZ, 0FP000Z, 0FP002Z, 0FP003Z, 0FT00ZZ, 0FT04ZZ, 0FT10ZZ, 0FT14ZZ, 0FT20ZZ, and 0FT24ZZ (*n* = 80,070). The exclusion criteria were as follows: (1) patients with missing data; (2) less than 18 years of age (Fig. 1). Ultimately, a total of 71,348 patients meeting the specified criteria and possessing comprehensive data were identified through the screening process.

Depending on the occurrence of POD, selected cases were divided into two groups. POD is defined by ICD-10-CM diagnostic code 4181, R4182, R4183, 4184, R41841, R41842, R41843, 41,844, R4189, F060, F062, F10121, F11921, F13921, F05, F11121.

The primary outcome of the study was POD. Surgery-related complications during hospitalization were collected from the National Inpatient Sample database by ICD-10-CM diagnostic code. Complications included sepsis, dementia, urinary retention, gastrointestinal complications, cardiac arrest, acute renal failure, pneumonia, continuous invasive mechanical ventilation, blood transfusion, respiratory failure, and acute myocardial infarction. Surgical complications included bile leakage, Intra-abdominal abscess, liver ascites, wound infection, and wound dehiscence / non-healing. If other comorbidities were identified in previous studies as important risk factors for any kind of POD, these comorbidities may be analyzed at the discretion of the authors.

### Data analysis

We began by examining the data distribution and calculating the basic descriptive statistics for all the variables included in the analysis. Afterwards, the continuous variable data were compared using a T-test, while the categorical data were compared using either χ2 test or Fisher exact test. To identify the factors that independently predict POD following hepatic resection, we conducted univariate and multivariate regression analyses. In order to evaluate the impact of various pre-surgery factors on the requirement for POD, we computed the adjusted odds ratio. These factors included patient characteristics like age, race, and teaching status of hospitals, as well as patient comorbidities and concurrent operations such as wound infection, hemorrhage, and pulmonary embolism. We used the R software (v3.5.3) to carry out all statistical analyses on the NIS database. Due to the large sample size, a P-value ≤ 0.001 was statistically significant.

## Results

### Occurrence of POD in hepatic resection cases

**Fig. 2:**
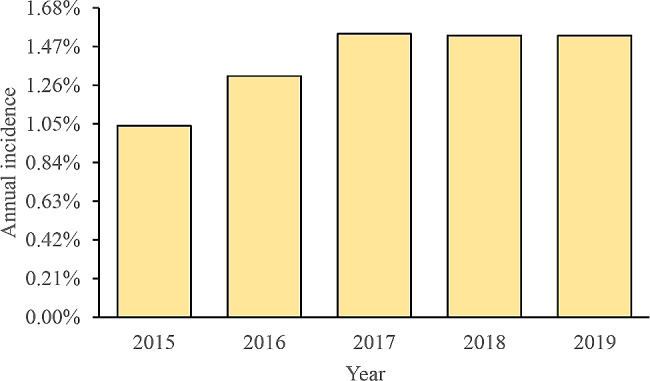
Annual incidence of postoperative delirium after hepatic resection

Patient demographics, hospital characteristics, and comorbidities are among the categories of variables outlined in Table 1. 71,348 hepatic resections were screened from the National Inpatient Sample database from 2015 to 2019. The incidence of POD was 1.46% (*n* = 1,039/71,348) (Table 2) and exhibited a growing trend from 2015 to 2019 (from 1.04 to 1.53%) (Fig. 2).

**Table 1:** Variables used in binary logistic regression analysis

Variables Categories	Specific Variables
**Patient demographics**	Age (≤ 64 years and ≥ 65 years), sex (male and female), race (White, Black, Hispanic, Asian or Pacific Islander, Native American and Other)
**Hospital characteristics**	Type of admission (non-elective, elective), location of the hospital (northeast, Midwest or north central, south, west), bed size of hospital (small, medium, large), type of insurance (Medicare, Medicaid, private insurance, self-pay, no charge, other), teaching status of hospital (nonteaching, teaching), Way of liver resection surgery (Laparoscopic, Open, Laparoscopic + Open)
**Comorbidities**	dementia, alcohol abuse, sleep apnea syndrome, pulmonary circulation disorders, deficiency anemia, Parkinson disease, valvular disease, congestive heart failure, hypertension, cerebrovascular diseases, depression, lymphoma, diabetes (uncomplicated), drug abuse, liver disease, fluid and electrolyte disorders, coagulopathy, metastatic cancer, other neurological disorders, peripheral vascular disorders, paralysis, psychoses, solid tumor without metastasis, obesity and weight loss
**Complications**	Sepsis, Acute myocardial infarction (AMI), Urinary retention, Gastrointestinal complication, Cardiac arrest, Acute renal failure, Pneumonia, Continuous invasive mechanical ventilation, Blood transfusion, Respiratory failure

**Table 2:** Patient characteristics and outcomes after hepatic resection (2015–2019)

Characteristics	POD	No POD	P
**Total (n = count)**	10,39	7,030,9	
**Total incidence (%)**	1.46		
**Age (median, years)**	67(58,76)	61(49, 70)	< 0.001
**Age group (%)**			
18–44	8.66	19.03	< 0.001
45–64	33.59	41.26	
65–74	27.05	23.61	
≥ 75	30.70	16.10	
**Gender (%)**			
Female	43.31	52.58	< 0.001
Male	56.69	47.42	
**Race (%)**			
**White**	71.32	66.52	0.007
**Native American**	0.38	0.60	
**Hispanic**	7.99	11.46	
**Black**	13.86	14.53	
**Asian or Pacific Islander**	2.98	3.38	
**Other**	3.46	3.51	
**Number of Comorbidity (%)**			
0	0.19	2.20	< 0.001
1	0.77	7.60	
2	3.67	14.84	
≥ 3	95.67	75.35	
**LOS (median, d)**	13 (8–23)	5 (3–9)	< 0.001
**Total cost (median, $)**	1,481,89 (7,745,2–3,274,52)	6,839,0.0 (4,166,3.5-1,182,83.0)	< 0.001
**Type of insurance (%)**			
Medicare	60.27	42.35	< 0.001
Self-pay	2.80	3,74	
Private insurance	21.31	35.79	
No charge	0.19	0.34	
Medicaid	12.54	15.11	
Other	2.89	2.66	
**Bed size of hospital (%)**			
Small	10.59	13.70	< 0.001
Medium	22.14	26.63	
Large	67.28	59.67	
**Elective admission (%)**	21.37	35.60	< 0.001
**Type of hospital (teaching %)**	88.61	81.94	< 0.001
**Region of hospital (%)**			
Northeast	22.43	19.52	0.004
West	19.54	19.37	
South	35.51	40.70	0.004
Midwest or North Central	22.52	20.41	
**Died (%)**	12.61	4.11	< 0.001
**Way of liver resection surgery(%)**			
Laparoscopic	29.32	10.68	< 0.001
Open	70.39	89.03	
Laparoscopic + Open	0.28	0.29	

POD: Postoperative Delirium, LOS: Length of stay

Bedsize assesses the number of short-term acute beds in a hospital. The hospital’s bedsize category is nested within location and teaching status

### Patient demographics

**Fig. 3:**
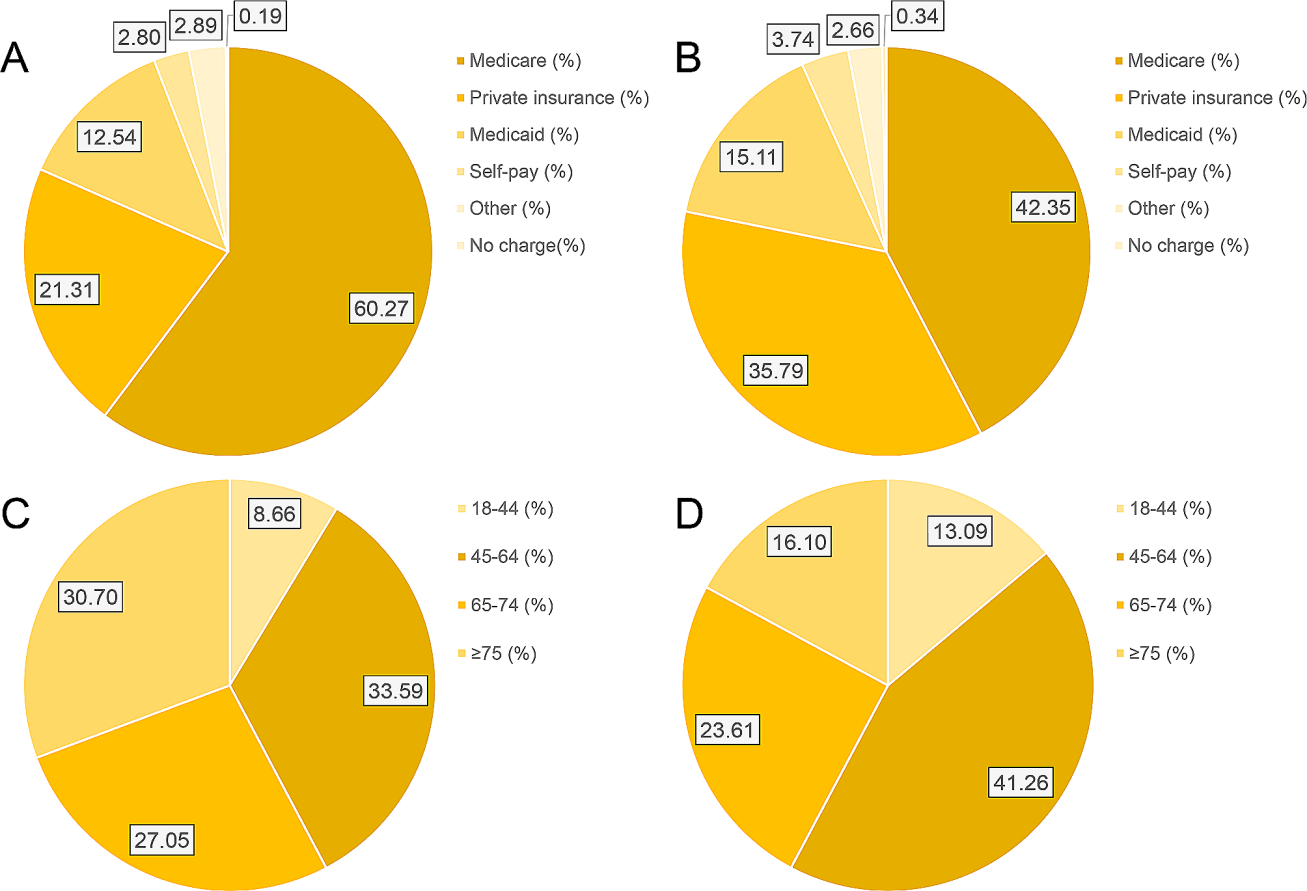
Patient demographics and hospital characteristics between the two surgical groups. **A** Type of insurance analysis of postoperative delirium patients. **B** Type of insurance analysis of patients without postoperative delirium. **C** Age distribution analysis of postoperative delirium patients. **D** Analysis of age distribution of patients without postoperative delirium

**Fig. 4:**
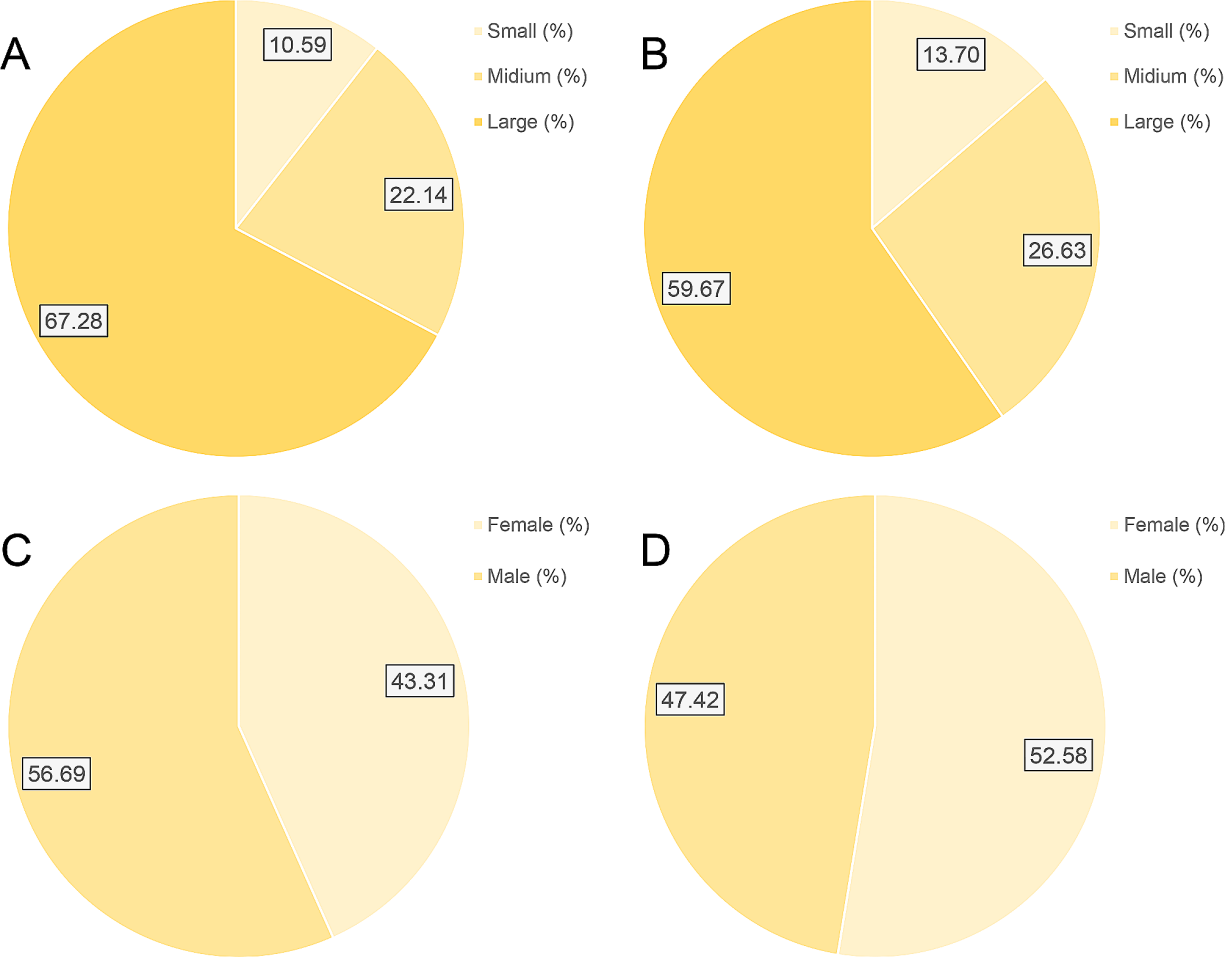
Patient demographics and hospital characteristics between the two surgical groups. **A** Racial distribution analysis of postoperative delirium patients. **B** Racial distribution analysis of patients without postoperative delirium. **C** Gender distribution analysis of postoperative delirium patients. **D** Gender distribution of patients without postoperative delirium

POD cases tended to be 6 years older (67 years vs. 61 years) and comprised 13.38% (56.69% vs. 43.31%) more males than unaffected populations (*P* < 0.001) (Table 2; Figs. 3C, D and 4C, D). Besides, the two groups exhibited comparable age distributions, and a lower occurrence of POD was observed among patients younger than 65 years (*P* < 0.001) (Table 2). Whites constituted the predominant race (nearly 66.59%) of the study population. However, the two groups were comparable in terms of race (*P* = 0.007) (Table 2).

### Hospital characteristics

Individuals who experienced POD following hepatic resection were 14.23% less likely to undergo elective admission compared to those who did not encounter such complications (35.60% vs. 21.37%, *P* < 0.001) (Table 2). Additionally, in-hospital POD was predominantly observed in teaching hospitals (88.61% vs. 88.91%, *P* < 0.001) (Table 2). Regarding hospital bed capacity, the occurrence of in-hospital POD was 3.18% higher in facilities categorized as Medium or large (24.30% vs. 27.48%, 67.28% vs. 59.67%, *P* < 0.001) (Table 2; Fig. 4A & B). In addition, the incidence of in-hospital POD was 78.35% higher with open hepatectomy (10.68% vs. 89.03%, *P* < 0.001) (Table 2). Besides, there was no significant difference in the hospital region between the two groups (*P* = 0.004) (Table 2).

### Adverse outcomes of POD after hepatic resection

Unsurprisingly, patients with POD had significantly three times higher in-hospital mortality compared with those with undiagnosed POD (12.61% vs. 4.11%; *P* < 0.001) (Table 2). POD cases had an 8-day longer hospitalization (13 vs. 5 days; *P* < 0.001) (Table 2). As a result, POD increases medical spending. The study found that the total hospitalization expenses were raised significantly by $79,799 after POD ($148,189 vs. $68,390, *P* < 0.001) (Table 2). In terms of the type of insurance, the proportion of individuals with private insurance in the POD group was lower by 14.48% (21.31% vs. 35.79%, *P* < 0.001) (Table 2; Fig. 3A & B). The number of comorbidities was significantly higher in patients with POD (95.67% vs. 75.35%, *P* < 0.001), which represented more comorbidities (Table 2).

### Risk factors of POD following hepatic resection

Logistic regression analysis was used to determine factors predisposing to POD (Tables 3 and 4), which included patients older than 65 years (OR = 1.30; CI = 1.22–1.39), teaching hospital (odds ratio [OR] = 1.85; CI = 1.52–2.25), Medium hospital bed size capacity (OR = 1.54; CI = 0.91–1.45), large hospital bed size capacity (OR = 1.64; CI = 1.34–2.02), depression (OR = 1.45; CI = 1.21–1.74), fluid and electrolyte disorders (OR = 1.27; CI = 1.11–1.47), coagulopathy (OR = 1.36; CI = 1.17–1.59), other neurological disorders (OR = 36.58; CI = 31.46–42.53), psychoses(OR = 1.77; CI = 1.34–2.34), weight loss(OR = 1.59; CI = 1.38–1.83).

**Table 3:** Risk factors associated with POD after hepatic resection

Variable	Multivariate Logistic Regression			
	OR	95% CI		*P*
**Age ≥ 65 years old**	1.30	1.22–1.39		<0.001
**Female**	0.79	0.70–0.90		<0.001
**Race**				
White	Ref	——		——
Native American	0.56	0.21–1.50		0.245
Hispanic	0.71	0.56–0.90		0.004
Black	0.90	0.75–1.08		0.259
Asian or Pacific Islander	0.80	0.55–1.15		0.228
Other	1.01	0.72–1.42		0.951
**Number of Comorbidity**				
1	1.12	0.24–5.28		0.886
2	2.39	0.57–9.94		0.232
≥ 3	10.86	2.71–43.57		0.001
**Type of insurance**				
Medicare	Ref	——		——
Self-pay	0.83	0.56–1.24		0.366
Private insurance	0.69	0.57–0.84		<0.001
No charge	0.66	0.16–2.67		0.556
Medicaid	0.86	0.68–1.09		0.226
Other	1.13	0.77–1.66		0.522
**Bed size of hospital**				
Small	Ref	——		——
Medium	1.54	0.91–1.45		0.223
Large	1.64	1.34–2.02		<0.001
**Elective admission**	0.61	0.52–0.71		<0.001
**Teaching hospital**	1.85	1.52–2.25		<0.001
**Region of hospital**				
Northeast	Ref	——		——
West	0.95	0.78–1.16		0.623
South	0.82	0.69–0.97		0.018
Midwest or North Central	0.88	0.74–1.07		0.195

POD: Postoperative delirium, OR: Odds ratio, CI: Confidence interval

**Table 4:** Relationship between POD and preoperative comorbidities

Comorbidities		Univariate Analysis			Multivariate Logistic Regression			
		**No POD**	**POD**	**P**		**OR**	**95% CI**	**P**
Preoperative comorbidities								
	Chronic blood loss anemia	1220(1.74%)	26(2.50%)	0.061		1.40	0.91–2.15	0.130
	Alcohol abuse	4760 (6.77%)	96 (9.24%)	0.002		0.88	0.69–1.11	0.270
	Sleep apnea syndrome	8963 (12.75%)	77 (7.41%)	<0.001		0.71	0.56–0.90	0.005
	Deficiency anemia	4791 (6.81%)	73 (7.03%)	0.788		0.93	0.72–1.21	0.595
	Parkinson disease	295 (0.42%)	15 (1.44%)	<0.001		1.72	1.00-2.97	0.051
	Valvular disease	2966 (4.22%)	60 (5.77%)	0.013		1.08	0.81–1.45	0.614
	Congestive heart failure	6597 (9.38%)	182 (17.52%)	<0.001		1.15	0.95–1.39	0.146
	Hypertension	40058 (57.97%)	622 (59.87%)	0.062		0.97	0.84–1.12	0.639
	Depression	8881 (12.63%)	174 (16.75%)	<0.001		1.45	1.21–1.74	<0.001
	Cerebrovascular diseases	691 (1.01%)	37 (3.60%)	<0.001		1.00	0.41–2.21	0.035
	Lymphoma	1209 (1.72%)	29 (2.79%)	0.009		0.79	0.53–1.19	0.256
	Diabetes, uncomplicated	11686 (16.62%)	131 (12.61%)	<0.001		1.00	0.82–1.22	0.998
	Drug abuse	1612 (2.29%)	33 (3.18%)	0.060		1.02	0.70–1.50	0.902
	Liver disease	30200 (42.95%)	443 (42.64%)	0.838		1.08	0.93–1.25	0.326
	Fluid and electrolyte disorders	22529 (32.04%)	621 (59.77%)	<0.001		1.27	1.11–1.47	0.001
	Coagulopathy	11048 (15.71%)	354 (34.07%)	<0.001		1.36	1.17–1.59	<0.001
	Metastatic cancer	25049 (35.63%)	443 (42.64%)	<0.001		1.01	0.86–1.19	0.892
	Other neurological disorders	4056 (5.77%)	770 (74.11%)	<0.001		36.58	31.46–42.53	<0.001
	Peripheral vascular disorders	3243 (4.61%)	81 (7.80%)	<0.001		1.24	0.96–1.60	0.094
	Paralysis	740 (1.05%)	38 (3.66%)	<0.001		1.07	0.75–1.53	0.694
	Obesity	19586 (27.9%)	162 (15.6%)	<0.001		0.77	0.64–0.92	0.005
	Psychoses	1936 (2.75%)	67 (6.45%)	<0.001		1.77	1.34–2.34	<0.001
	Weight loss	12938 (18.40%)	430 (41.39%)	<0.001		1.59	1.38–1.83	<0.001
	Solid tumor without metastasis	22907 (32.58%)	394 (37.92%)	<0.001		1.27	1.09–1.47	0.002
	Pulmonary circulation disorders	1860 (2.64%)	41 (3.95%)	0.010		0.98	0.69–1.40	0.918

POD: Postoperative delirium, OR: Odds ratio, CI: Confidence interval

### Additional comorbidities and complications associated with POD following hepatic resection

Univariate analysis showed that POD cases experienced more perioperative complications during hospital stay, encompassing sepsis, dementia, acute myocardial infarction, urinary retention, gastrointestinal complication, cardiac arrest, acute renal failure, pneumonia, continuous invasive mechanical ventilation, blood transfusion, respiratory failure, intra-abdominal abscess, liver ascites, wound infection, wound dehiscence/non-healing (*P* < 0.001) (Table 5). Multivariate analysis revealed that POD after hepatic resection may be associated with perioperative complications: sepsis (OR = 1.61; CI = 36-1.91), dementia(OR = 3.46; CI = 2.74–4.37), urinary retention (OR = 1.80; CI = 1.43–2.34), gastrointestinal complication (OR = 1.70; CI = 1.23–2.35), acute renal failure (OR = 1.89; CI = 1.65–2.17), pneumonia (OR = 1.43; CI = 1.19–1.73), continuous invasive mechanical ventilation (OR = 1.82; CI = 1.33–2.49), blood transfusion (OR = 1.62; CI = 1.37–1.89), respiratory failure (OR = 1.50; CI = 1.19–1.9), wound dehiscence/ non-healing (OR = 2.53; CI = 1.57–4.09).

**Table 5:** Relationship between POD and postoperative complications

**Complications**	**Univariate Analysis**			Multivariate Logistic Regression		
	No POD	POD	P	OR	95% CI	P
**Medical complications**						
Sepsis	6388 (9.09%)	269 (25.89%)	<0.001	1.61	1.36–1.91	<0.001
Dementia	1254 (1.78%)	98 (9.43%)	<0.001	3.46	2.74–4.37	<0.001
Urinary retention	2246 (3.19%)	74 (7.12%)	<0.001	1.83	1.43–2.34	<0.001
Gastrointestinal complication	1366 (1.94%)	56 (5.39%)	<0.001	1.70	1.23–2.35	0.001
Cardiac arrest	478(0.68%)	25 (2.41%)	<0.001	1.67	1.08–2.54	0.020
Acute renal failure	13321 (18.95%)	456(43.89%)	<0.001	1.89	1.65–2.17	<0.001
Pneumonia	4322 (6.15%)	161 (15.50%)	<0.001	1.43	1.19–1.73	<0.001
Continuous invasive mechanical ventilation	1181 (1.68%)	48(4.62%)	<0.001	1.82	1.33–2.49	<0.001
Blood transfusion	6627 (9.43%)	222 (21.37%)	<0.001	1.62	1.37–1.89	<0.001
Respiratory failure	2220 (3.16%)	98 (9.43%)	<0.001	1.50	1.19–1.9	<0.001
Acute myocardial infarction	332 (1.06%)	167 (2.79%)	<0.001	1.23	0.83–1.83	0.413
**Surgical complications**						
Bile Leakage	65 (0.09%)	3 (0.29%)	0.042	1.67	0.49–5.54	0.420
Intra-abdominal abscess	2294 (3.26%)	63 (6.06%)	<0.001	1.02	0.77–1.35	0.883
Liver ascites	2294 (3.26%)	63 (6.06%)	<0.001	1.05	0.80–1.39	0.715
Wound infection	368 (0.52%)	23 (2.21%)	<0.001	1.76	1.09–2.85	0.021
Wound dehiscence/non-healing	260 (0.37%)	22 (2.12%)	<0.001	2.53	1.57–4.09	<0.001

POD: Postoperative delirium, OR: Odds ratio, CI: Confidence interval

## Discussion

Postoperative delirium often presents 24 to 96 h after surgery and presents with acute confusion, disorganized attention, and decreased perception of the environment, with symptoms that may or may not be related to organic disease and usually resolves during hospitalization [20]. It is estimated that about 4–61% of patients develop POD after surgery [21], which may lead to a cascade of adverse outcomes, such as lengthy hospitalization, loss of functional independence, and cognitive decline [6, 11, 22]. This investigation presents an extensive health-economic analysis of delirium following hepatic resection. Over the period spanning 2015 to 2019, the incidence of POD exhibited an upward trend, rising from approximately 1.04–1.53%. The comparatively low rates observed in 2015 may be attributed to the implementation of the ICD-10-CM coding system (Fig. 2). Our study revealed an overall occurrence of 1.46% following hepatic resection, significantly lower than the rates reported in the literature, which ranged from 8.4–22.4% [17, 23, 24]. Several possible reasons can also explain this apparent discrepancy. First, limitations of the National Inpatient Sample database, such as providing only in-hospital POD, high specificity (low false-positive rate), and low sensitivity (high false-negative rate), might result in an underestimation of the actual rates [19]. Secondly, the definition and diagnosis of delirium varies from agency to institution, depending on the criteria employed, which can also contribute to this discrepancy [16, 25, 26]. Third, the previous literature has observed a small and selective presence in older patients, resulting in a high incidence [17, 18, 24]. Fourth, differences in study design interpretation and diagnostic criteria could potentially account for the difference in the documented occurrence of postoperative delirium, whose clinical diagnosis can be prone to oversight [27]. Accordingly, larger, prospective case-control studies are warranted to understand the broader picture.

Advanced age is a well-known risk factor for POD [17, 18]. In accordance with previous reports, our findings revealed that advanced age was a robust predictor of POD after hepatic resection. Patients with POD tended to be 6 years older than those who were not affected. In addition, in terms of age distribution, patients over 65 years of age make up a larger proportion of the POD group. A potential explanation for this observation is that age-related factors, such as brain atrophy, diminished neuron density, decreased blood flow, and neurotransmitter level reductions, have been demonstrated to render the elderly population particularly susceptible to delirium [27, 28]. Additionally, we found that a smaller proportion of females were present in patients with POD, which was also found to be protective during logistic regression.

As expected, patients who underwent hepatic resections during elective surgery were associated with lower rates of POD, given that most patients admitted for elective surgery are in good health or have been adequately evaluated and prepared prior to surgery [29]. This finding was further confirmed by the finding that elective admission was a protective factor for POD. One possible reason why teaching hospitals are a predictor of in-hospital POD risk is that cases in these hospitals often have complex complications [30]. In addition, laparoscopic hepatectomy has developed rapidly [31], and many studies have shown that laparoscopic hepatectomy is safer and more feasible than open hepatectomy [31, 32]. In particular, the postoperative recovery was faster in the laparoscopic hepatectomy group than in the open hepatectomy group. These results are further validated by our findings that the incidence of in-hospital POD is significantly reduced with laparoscopic hepatectomy. In terms of the hospital bed capacity, hospitals categorized as medium or large capacity correlated with increased rates of POD, although the cause is unclear and may be due to multiple factors.

Several studies on POD after hepatic resection have shown that risk stratification, pre-screening, and optimal care are essential to improve prognosis [17, 18, 24]. Therefore, in order to prevent the occurrence of POD, emphasis should be placed on comprehending the preoperative risk factors. Logistic regression analysis results were consistent with the literature; the OR value was highest for psychosis due to other neurological disorders before surgery (36.58) (Table 4), indicating that these medical comorbidities were strongly correlated with POD and should be addressed preoperatively. Patients with a history of other neuropsychiatric disorders, such as depression (OR = 1.45) and psychoses (OR = 1.77), were identified as having an elevated risk of developing POD [17, 33]. Other comorbidities such as fluid and electrolyte disorders (OR = 1.27), coagulopathy (OR = 1.36), weight loss (OR = 1.59), and coagulopathy (OR = 1.53) had been documented as factors predisposing to POD [33, 34]. Interestingly, female (OR = 0.79), elective admission (OR = 0.61), and Private insurance (OR = 0.69) yielded a protective effect against POD, although the reasons are unknown and could be multifaceted. Several previous findings may be relevant and worth discussing. Melatonin is a hormone released by the pineal gland and associated with female hormones [34]. Previous studies have documented that changes in melatonin metabolism or disrupted circadian patterns may participate in POD pathogenesis [35].

Consistent with previous studies [36, 37], our data analysis showed that patients with POD had a median length of stay of six days longer and an increase in total hospital costs of $79,799 compared to patients who did not develop POD. POD may be associated with perioperative complications (sepsis, dementia, urinary retention, gastrointestinal complications, acute renal failure, pneumonia, continuous invasive mechanical ventilation, blood transfusion, respiratory failure) (Table 5). Besides, people with POD often have impairments in consciousness, perception, or cognitive function and are unable to follow guidance for care and rehabilitation [33, 38]. Patients with POD are more often paid through Medicare than those without POD. In addition, private insurance is a protective factor for POD, underscoring the significant influence of medical insurance type in this context. As a result, the increase in complications can result in more than three times the in-hospital mortality rate in delirium cases compared to those who are not affected. Therefore, the number of comorbidities was significantly higher in patients with POD since a higher number of comorbidities is indicative of poorer preoperative general conditions, and more comorbidities may contribute to heightened postoperative complications comprising delirium.

Although certain preoperative risk factors like advanced age, psychosis, depression, and neurological disorders are non-modifiable, it is beneficial for clinicians to engage in consultations with patients, obtain informed consent, and proactively manage those identified as being at a heightened risk of POD. Other factors predisposing to POD, including coagulopathy, weight loss, and fluid and electrolyte imbalances, are amenable to modification to some extent. Patients presenting with one or more of these comorbidities can undergo optimization to enhance their readiness for elective procedures. This optimization may involve interventions such as transfusions for patients with coagulopathy and effective management of fluid and electrolyte balance. Consequently, these findings hold significant importance and offer potential benefits in the treatment of POD following hepatic resection [33, 39]. Furthermore, Sensory deficit correction, it encourages the use of correctors and technical aids such as glasses, hearing aids, and dentures, among others; environmental management, installation of a clock and other orientation elements in the patient’s room to promote orientation, in addition to minimizing environmental stressors; sleep protocol, lowering of lights, noise, and administration of nighttime drugs; hydration protocol, monitoring of the patient’s hydration and access to it; and reduction of medication, which have proven to be highly effective in preventing POD [40, 41].

Our study offers notable strengths, encompassing a substantial sample size, national representativeness, and the application of multivariate regression models to mitigate confounding variables. However, it is imperative to recognize inherent shortcomings associated with the use of the National Inpatient Sample database. Firstly, the collection of each case data was confined to the duration of their hospital stay, thereby omitting complications or outcomes post-discharge, including readmission rates and long-term follow-up data from this database. Since only early-stage hospitalized cases are included, this limitation may lead to an underestimation of the incidence of POD [19, 23, 24]. Secondly, akin to any extensive administrative dataset, coding, and documentation discrepancies or misclassifications may occur [16, 19]. In addition, the evaluation is constrained to variables documented in the NIS database. Notably, the relevant surgical types and anesthetic factors that could potentially influence POD, such as no record of the duration of surgery, size of liver cancer, and invasion, and some indications for surgery (hepatocellular carcinoma, intrahepatic cholangiocarcinoma, metastatic cancer, and Others.) are not strictly defined, operation duration, anesthesia duration, anesthesia mode, and the selection of anesthetic agents, were not captured in the NIS database [17, 18]. This was a weakness of this study, but it did not affect the analysis of risk factors. The advantage of the NIS database is that the sample size is larger and the data is more convincing. The results of this study provide a better treatment strategy for the development of liver resection.

## Conclusions

POD that occurs after hepatic resection is a costly complication, with an overall incidence of 1.46%. From 2015 to 2019, the annual incidence of POD gradually increased. This study identified several risk factors for POD after hepatic resection, including teaching hospitals, non-selective admissions, older male patients, depression, fluid and electrolyte disorders, coagulation disorders, and weight loss. In addition, POD is associated with sepsis, dementia, urinary retention, gastrointestinal complications, acute renal failure, pneumonia, continuous invasive mechanical ventilation, blood transfusion, respiratory failure, and wound dehiscence / non-healing. Young female patients, elective admissions, and private insurance were identified as protective factors. The incidence of POD following hepatic resection surgery is associated with higher total hospitalization costs, longer LOS, and higher in-hospital mortality.

## Data Availability

The datasets are available at https://www.ahrq.gov/data/hcup/index.html.

## Abbreviations

POD: Postoperative Delirium
ICD: International Classification of Diseases
NIS: National Inpatient Sample
ICU: Intensive Care Unit
LOS: Length of Stay
OR: Odds ratio
CI: Confidence interval
